# Improving the Efficiency and Orthogonality of Genetic Code Expansion

**DOI:** 10.34133/2022/9896125

**Published:** 2022-06-06

**Authors:** Xian Fu, Yijian Huang, Yue Shen

**Affiliations:** ^1^BGI-Shenzhen, Shenzhen 518083, China; ^2^Guangdong Provincial Key Laboratory of Genome Read and Write, Shenzhen 518120China; ^3^School of Future Technology, University of Chinese Academy of Sciences, Beijing 100049, China; ^4^Shenzhen Institute of Synthetic Biology, Shenzhen Institutes of Advanced Technology, Chinese Academy of Sciences, Shenzhen 518055, China

## Abstract

The site-specific incorporation of the noncanonical amino acid (ncAA) into proteins via genetic code expansion (GCE) has enabled the development of new and powerful ways to learn, regulate, and evolve biological functions *in vivo*. However, cellular biosynthesis of ncAA-containing proteins with high efficiency and fidelity is a formidable challenge. In this review, we summarize up-to-date progress towards improving the efficiency and orthogonality of GCE and enhancing intracellular compatibility of introduced translation machinery in the living cells by creation and optimization of orthogonal translation components, constructing genomically recoded organism (GRO), utilization of unnatural base pairs (UBP) and quadruplet codons (four-base codons), and spatial separation of orthogonal translation.

## 1. Introduction

**Figure 1:**
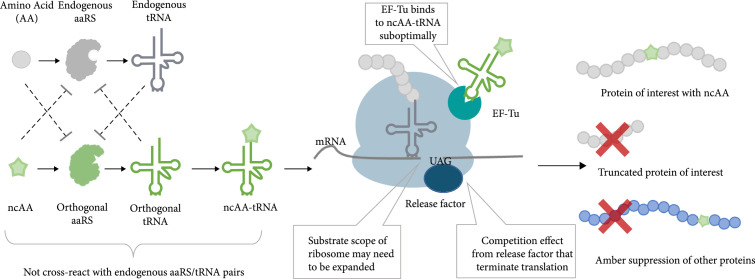
Schematic diagram showing the principle and inherent problems of the amber codon-mediated genetic code expansion. The ncAA (green star) is ligated to the amber suppressor tRNA by its cognate aminoacyl–tRNA synthetase (aaRS). The introduced orthogonal aaRS/tRNA pair (green) decodes amber codon on mRNA to site-specifically incorporate ncAA into the protein of interest. The orthogonal aaRS/tRNA pair does not cross-react with endogenous tRNAs, amino acids (AAs), and aaRSs (shown in grey). The key problems of the amber codon-directed ncAA insertion by the orthogonal aaRS/tRNA pair are listed, including the competition from release factor, suboptimal interaction between elongation factor Tu (EF-Tu) and ncAA-tRNA, and restricted substrate scope of ribosome. The amber codon-mediated genetic code expansion would produce the protein of interest containing ncAA at the target site as well as the undesired products including the truncated POI and other C-terminally extended proteins.

Genetically incorporating ncAA with diverse functional groups into a protein of interest (POI) by GCE technology is a powerful method to manipulate protein functions and enables many applications, including the development of new drugs, biopolymers, and novel probes, as well as investigation of protein posttranslational modifications (PTMs). To date, over 200 ncAAs have been cotranslationally incorporated into POI in living cells by GCE approaches [1]. Correct incorporation of ncAA by GCE requires orthogonal translational systems. As aminoacyl-tRNA synthetase and tRNA (aaRS/tRNA) pair plays the central role in ensuring accurate genetic code interpretation by attaching the appropriate amino acid onto its corresponding tRNAs, creating orthogonal aaRS/tRNA pairs is required for specific ncAA incorporation. The orthogonality means the introduced aaRS/tRNA pair should not cross-react with endogenous aaRS/tRNA pairs (Figure 1). The anticodon of orthogonal tRNA is then engineered to pair with a “blank” codon that is not assigned to a canonical amino acid. The amber stop codon (TAG) is most often used. Finally, the amino acid binding pocket of orthogonal aaRS is modified to recognize the desired ncAAs and reject canonical amino acids selectively. Thus, by introducing the orthogonal aaRS/tRNA pair and hijacking the host translational apparatus, ncAAs can be site-specifically incorporated into proteins in response to the amber stop codon *in vivo* (Figure 1). With orthogonal aaRS/tRNA pairs, reprogramming the genetic code for ncAA insertion is now possible in bacteria and eukaryotic cells and animals [2, 3].

Despite the rapid progress of our ability to genetically incorporate diverse ncAAs via GCE, two inherent problems of ncAA incorporation in living cells, including ambiguous decoding due to the lack of a specific blank codon and low incorporation efficiency, need to be addressed to ensure further practical and sophisticated applications (*e.g*., the mass and accurate production of ncAA-containing proteins, especially in eukaryotes). Since each of the 64 triplet codons is utilized in every organism to synthesize natural proteins, the codon chosen for ncAA incorporation by GCE has on two definitions in the same cell, which may cause serious problems. Taking the TAG-directed ncAA incorporation as an example, ambiguous decoding of the amber codon causes two issues. First, introducing orthogonal aaRS/tRNA_CUA_ pair gives rise to global suppression of all TAG codons, resulting in impaired physiological function [4]. Second, release factors compete with the introduced aaRS/tRNA pair, resulting in a portion of polypeptide synthesis terminating at the amber codon [5] (Figure 1). In addition, the introduced aaRS/tRNA pair often does not perfectly fit with the endogenous translational machinery. For instance, the orthogonal tRNA might interact with the elongation factor poorly and the endogenous ribosome might function as a suboptimal decoder for the ncAA-charged tRNA [3, 6]. Thus, it is pivotal to improve the efficiency of GCE by systematically optimizing different steps involved in the biosynthesis of ncAA-containing protein (Figure 1).

In this review, we provide essential background on aforementioned problems of ncAA incorporation in living cells and focus primarily on recent efforts and achievements overcoming many challenges for genetically encoding noncanonical biopolymers. We also compared distinct strategies for improving the orthogonality and efficiency of GCE, including the advantages and disadvantages, the improved efficiency, and different methods utilized in these studies. This review does not cover the advances in GCE utilizing *in vitro* protein synthesis [7] and many exciting applications of GCE [8].

## 2. Orthogonal Codons for ncAA Incorporation

**Figure 2:**
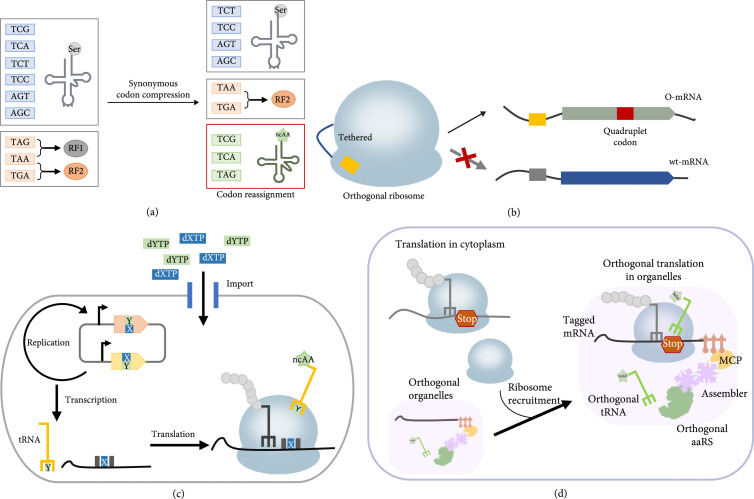
Different strategies to create orthogonal codons for ncAA incorporation. (a) Construction of genomically recoded *E. coli* to reassign two serine codons and the amber stop codon to ncAAs. The created blank codons are in the red box. (b) Quadruplet codon decoding by the orthogonal ribosome. The orthogonal ribosome specifically translates O-mRNA by base pairing between the engineered anti-SD sequence and complementary SD sequence in the 16S rRNA and O-mRNA, respectively (yellow box). The 50S and 30S subunit could be tethered to generate entirely orthogonal ribosomes. The ncAA is inserted into a protein of interest (POI) in response to a quadruplet codon (red box). (c) A semisynthetic organism could intake and retain an unnatural base pair (UBP) composed of dXTP and dYTP. X and Y denote NaM and TPT3, respectively. The dXTP and dYTP could be imported into *E. coli* cytosol by overexpression of a special nucleotide transporter. UBP could be transcribed into mRNAs and tRNAs containing unnatural codons and anticodons, respectively. The ncAA could be site-specifically incorporated into a POI response to the unnatural codon. (d) Orthogonally translating organelles enables mRNA-specific translation. Orthogonal translating organelles form a micron-sized structure in cells that consist of an mRNA-targeting system, an orthogonal aaRS/tRNA pair (green), the phase-separating protein (assembler). Assembler (purple) is fused to PylRS (green) and MCP (yellow). The ms2-tagged mRNA (ms2 is labeled in pink) bounds to MCP. Therefore, recruited ribosomes translate the tagged mRNA with an expanded genetic code, producing ncAA-containing proteins, whereas the standard genetic code is executed in the cytoplasm.

An ideal GCE requires a blank codon assigned explicitly to a ncAA. To fulfill the potential of GCE applications in living cells, creating chassis with orthogonal codons is highly desired. Here, we discussed several fundamental strategies to avoid ambiguous decoding by codon compression, creating additional codons, including quadruplet codons and UBPs, and codon reassignment of tagged mRNA by using orthogonally translating organelle (Figure 2). In addition, we also compared the advantages and disadvantages for these different strategies (Table 1).

**Table 1:** Comparing distinct strategies of creating orthogonal codons for ncAA incorporation.

Strategies	Organisms	Advantages	Disadvantages or bottleneck
Codon compression	*E. coli* [5, 12–14] *S. cerevisiae* [10]	(a) Fully orthogonal codon to eliminate the competition effect of release factors or ambiguous decoding by endogenous tRNAs	(a) A daunting and costly task to recode the whole genome of an organism, especially for eukaryotes (b) The GRO often has growth defect (c) Requiring case by case design and construction for different GRO

Quadruplet codons	*E. coli* [17, 19, 20, 22]	(a) A total of 256 blank codons could be generated in principle (b) No competition from the release factor	(a) Occurring +1 frameshift would enhance the misreading of the proteome (b) It is challenging to engineer the natural ribosome and to generate aaRS/tRNA pairs efficiently decoding the quadruplet codons (c) Not all quadruplet codons could be efficiently decoded till now

UBP	*E. coli* [26, 32, 33, 36] CHO cells [35]	(a) Generation of additional 152 blank codons by expanding the genetic alphabet from four letters to six letters (b) Highly orthogonal with minimal competition from the endogenous translational systems	(a) It is challenging to construct a variety of semisynthetic organisms that are able to intake and retain UBP and to store and retrieve increased genetic information (b) Only a subset of unnatural codons could be efficiently used for ncAA incorporation

Orthogonally translating organelles	HEK293T cells [39, 40]	(a) Spatially enriching the key components of the GCE machinery to minimize the ambiguous decoding (b) Requiring only rational design for aaRS and mRNA, without extensive directed evolution of other translational components (c) Reuse of the same TAG codon for distinct ncAA incorporation in different film-like organelles	(a) The strategy of using specialized organelles is limited to eukaryotic cells (b) The long-term impact of the synthetic organelles on cell physiology remains unknown (c) It is hard to quantify the incorporation efficiency of distinct ncAA in POI

### 2.1. Codon Compression by Genome Synthesis

As the genetic code is degenerate, construction of a GRO in which synonymous codons replace all target codons in the whole genomes is a fundamental way to generate blank codons (Figure 2(a)). Development of MAGE and conjugative assembly genome engineering (CAGE) methods allows genome-wide codon replacement [9]. A GRO designated as C321.*Δ*A, in which all known TAG codons in *E. coli* MG1655 have been substituted by TAA, was constructed in 2013 [5]. By removing the RF1, the amber stop codon in the C321.*Δ*A strain is completely assigned to ncAAs by expressing UAG-reading aaRS/tRNA pairs [5]. Similarly, the design of the ongoing synthetic yeast genome project includes TAG/TAA stop-codon swaps in all sixteen chromosomes [10]. The reassignment of sense codons to ncAAs could also be implemented by genome-wide substitution of a subclass of sense codons by their synonymous codons, followed by removing the corresponding tRNAs. Since sense codons are much higher frequent than stop codons in the genome, synthesizing entire recoded genomes is preferred over multiple-site editing such as MAGE and CRISPR–Cas9, which would likely introduce off-target mutations [5, 11]. Serine and leucine codons are chosen for codon compression in *E. coli* as the anticodon of tRNA^Ser^ and tRNA^Leu^ is not recognized as a tRNA identity element. Thus, the introduction of blank codon decoding tRNAs would not crosstalk with endogenous aaRSs. An ambitious project that is aimed at synthesizing 57-codon *E. coli* genome in which total seven codons (six sense codons and amber stop codon) were substituted for synonymous alternatives has been partially completed, highlighting the feasibility of drastically changing the genetic code [12]. Recently developed methods such as replicon excision-enhanced recombination (REXER) and genome stepwise interchange synthesis (GENESIS) facilitate the replacement of genomes with synthetic DNA [13], leading to the construction of syn61, the first entirely synthesized *E. coli* with a 61-codon genome [14]. A subsequent work shows that replacing two serine codons and TAG codon enables elimination of cognate tRNAs and RF1 in a single strain. The three blank codons can be assigned to three distinct ncAAs in the optimized syn61 strain expressing three mutually orthogonal aaRS/tRNA pairs [15] (Figure 2(a)).

### 2.2. Quadruplet Codons and Orthogonal Ribosome

In principle, a total of 256 quadruplet codons could be potentially explored for GCE. Quadruplet codon suppression by tRNA with a nucleotide extension in the anticodon was observed in nature [16]. Inspired by the naturally occurring +1 frameshift suppressors, quadruplet codon-mediated GCE has been developed for ncAA incorporation [17, 18]. In response to quadruplet codons, GCE confronts two major problems: (i) the natural ribosome decodes quadruplet codons poorly and (ii) expression of quadruplet codon-reading tRNAs would lead to proteome-wide misincorporation. By assessing structure-guided libraries in the decoding center, an orthogonal ribosome termed ribo-Q1 has been developed that efficiently translates quadruplet codons on its cognate mRNA termed O-mRNA (Figure 2(b)) [19]. The O-mRNA contains modified Shine-Dalgarno (SD) sequence and therefore is not recognized by native ribosomes. As ribo-Q1 was derived from ribo-X, an engineered ribosome that efficiently decodes TAG codon [20], both amber stop codon and quadruplet codons could serve as blank codons for ribo-Q1-mediated incorporation of multiple ncAAs on an O-mRNA [19, 21]. A recent study significantly improved the efficiency of orthogonal ribosome-mediated translation by optimizing O-mRNA sequences using thermodynamic models and algorithms, which resulted in simultaneous incorporation of four different ncAAs into a single protein directed by four different quadruplet codons [22].

As mentioned above, engineering the anti-SD sequence in the 16S rRNA together with the complementary SD sequence in O-mRNA could generate orthogonal 30S subunits that specifically translate O-mRNA (Figure 2(b)). However, the orthogonality relying on the small subunit is limited as the association between large subunits and the native, and orthogonal 30S subunits are stochastic. This limitation could be addressed by the creation of an orthogonal ribosome with tethered subunits (Ribo-T), which could work independently from the native ribosomes that are responsible for biosynthesis of endogenous proteins [23, 24] (Figure 2(b)). A Ribo-T variant with improved properties was recently evolved and could be harnessed to produce a green fluorescent protein that contains ncAAs at multiple sites [25].

### 2.3. Creating Additional Codons Using the Unnatural Base Pair

In addition to quadruplet codons, developing synthetic nucleotides that pair to form an UBP is another powerful way to create orthogonal codons for GCE. Pioneering studies have identified and developed several UBPs formed by noncovalent interactions, including hydrogen bonds and hydrophobic and packing interactions [26]. Some of these UBPs could be efficiently amplified by PCR [27, 28], selectively transcribed to RNA [29], and utilized to incorporate ncAAs via *in vitro* translation [30, 31]. A landmark work created a semisynthetic organism (SSO) bearing an expanded genetic alphabet. The SSO is able to intake and retain one class of UBP (dTPT3/dNaM and d5SICS/dNaM pairs) in a plasmid [32]. By optimizing the transporter to uptake the synthetic triphosphates and exploring the CRISPR-Cas9 system to prevent UBP loss, the SSO could stably retain the expanded genetic alphabet over 100 cell divisions [33]. To demonstrate that the dNaM/dTPT3 pair could be used as additional codons to retrieve increased genetic information, a further study showed that DNA containing dNaM (dX) and dTPT3 (dY) could be used as other codons to transcribe into mRNAs and tRNAs containing unnatural codons and anticodons, respectively. These unnatural codons could direct site-specific ncAA incorporation [34] (Figure 2(c)). Using a similar strategy, the unnatural codon-directed ncAA incorporation was demonstrated in eukaryotic cells [35]. In theory, the numbers of available codons would increase by 152 via expanding the genetic alphabet from four letters to six letters. A recent study that systematically screened the unnatural functional codon in SSOs identified additional unnatural codons that could efficiently produce ncAA-containing proteins [36]. By utilizing mutually orthogonal aaRS/tRNA pairs and tRNA^Ser^ (AYC) with endogenous seryl-tRNA synthetase, the SSO could incorporate two distinct ncAAs and serine into a POI in response to three different unnatural codons [36].

### 2.4. Orthogonally Translating Organelles

The blank codons are not required if the orthogonal translation could decode a specific codon only for the gene of interest in a spatially confined microenvironment. Phase separation is recently recognized as a common mechanism for accumulating high local concentrations of biomacromolecules such as proteins and RNAs [37, 38]. Inspired by phase separation, membrane-less orthogonal translating organelles targeting to microtubule plus-ends have been created that consists of an mRNA-targeting system (mRNA::ms2 fusion), a PylRS/tRNA^Pyl^ pair derivative, and the assembler that brings tagged mRNA into proximity of the orthogonal aaRS/tRNA pair (Figure 2(d)) [39]. Thus, cellular ribosomes near the organelle function with highly concentrated PylRS/tRNA^Pyl^ pair to translate the ms2-tagged mRNAs and produce ncAA-containing proteins [39]. The following study developed several orthogonal translating organelles that support GCE on surfaces of plasma, endoplasmic reticulum, Golgi, and mitochondrial membranes [40]. Remarkably, these dual film-like organelles compromising spatially orthogonal aaRS/tRNA pairs and tagged mRNA could use the same TAG codon for distinct ncAA incorporation, generating a eukaryotic cell with two expanded genetic codes [40].

## 3. Improving the Efficiency of ncAA Incorporation by GCE

Many factors may affect the efficiency of GCE, including the cellular concentration of ncAA [41], the expression level of introduced aaRS/tRNA pair in heterogeneous cells [42], codon context effects [43], the ligation efficiency of ncAA to a dedicated tRNA catalyzed by the corresponding aaRS [44], the compatibility of ncAA-tRNA with the elongation factor [45, 46], the efficiency of tRNA decoding by codon-anticodon pairing and peptide bond formation in the ribosome [47], and the competition effect from release factors that terminate polypeptide formation [48]. Increasing evidence suggests that many of the factors mentioned above are interrelated and have combined effects. Thus, efficient ncAA incorporation in the living cell requires optimization of expression and activity of the introduced aaRS/tRNA pair and systematically engineering many parts of the translational apparatus involved in many steps of protein synthesis. In this section, we summarized a series of efforts to improve the efficiency of ncAA incorporation by engineering of distinct translation components including aaRS/tRNA pairs, the elongation factor, the release factors, and the ribosome, with a particular focus on the relevant technologies and the improved efficiency (Table 2).

**Table 2:** Engineering translation components to improve the efficiency of ncAA incorporation.

Classification	Components	Blank codons	Host	Strategies	Improved performance
The aaRS/tRNA pair	Chimeric aaRS/tRNA	UAG	*E. coli*, mammalian cells	Rational design of chimeric aaRS/tRNA pairs by transplanting the key sequences from PylRS/tRNA^Pyl^	The chPheRS-1 showing higher activity than PylRS and *Mj*TyrRS systems in response to a single UAG codon based on the yield of purified protein and GFP reporter assay [53]
	aaRS/tRNA	UAG	*E. coli*	Computational analysis and new method to determine *in vivo* aminoacylation status, designated as tREX	The discovered and further evolved *Af*TyRS/tRNA^Tyr^ pair showing an efficiency 5-fold higher than derivatives of the *Mj*TyrRS/tRNA^Tyr^ pair [54]
	*Mj*TyrRS, chPylRS	UAG	*E. coli*	Evolved chPylRS and *Mj*TyrRS variants through PACE	The chPylRS variant with 9.7-fold improvement in the yield of proteins containing ncAA; the *Mj*TyrRS variant with >23-fold higher specificity to *p*-IF than *p*-NF [56]
	chPylRS	UAG	*E. coli*	Evolved PylRS variants through PANCE	PylRS variant with ~10-fold improvement in catalytic efficiency, as measured by $K_{\text{cat}}/K_{\text{m}}$ [57]
	*Mj*TyrRS	UAG	*E. coli*	Evolved *Mj*TyrRS variants through MAGE	Chromosomally integrated variants with up to 25-fold increased yield for producing proteins containing pAcF and pAzF [58]
	*Mb*tRNA^Pyl^	UAG	*S. cerevisiae*	Promoter optimization of tRNA^Pyl^	With similar incorporation efficiency compared to *Ec*TyrRS/tRNA^Tyr^ pair in yeast for producing human superoxide dismutase [60]
	tRNA^Pyl^	UAG	Mammalian cells	Rational design to optimize tRNA^Pyl^ compatibility	The best tRNA^Pyl^ variant showing 2.5-fold increase in suppression efficiency [61]
	*Mm*tRNA^Pyl^	UAG	Mammalian cells	Optimization of tRNA^Pyl^ expression using external promoters and increased gene copies	Production of BocK-containing protein in an amount accounting for 1% of the total proteins in HEK cells [62]
	tRNA^Pyl^	UAG	*E. coli*	Rationally designed small libraries for the acceptor stem and T stem of tRNA^Pyl^	~3-fold and a 5-fold increase in AcK incorporation in response to one and two UAG sites, respectively [64]
	*Ec*tRNA^Tyr^ *Ec*tRNA^Leu^	UAG	*S. cerevisiae*	Optimization of *Ec*tRNA^Tyr^ in yeast using a Pol III promoter that contains A- and B-box	GFP reporter assay shows 9-fold increase in suppression efficiency by using SNR52 promoter [107]

Elongation factor	EF-Tu	UAG	*E. coli*	Structure-guided libraries of the amino acid-binding pocket	Production of 25 *μ*g/L MBP-MEK1 (Sep^218^, Glu^222^) and 1 *μ*g/L MBP-MEK1 (Sep^218^, Sep ^222^) [71]
	EF-Tu	UAG	*E. coli*	Successive rounds random mutation of key residues in the amino acid-binding pocket	Production of 3 mg/L H3S10ph [73]
	EF-Tu	UAG	*E. coli*	Structure-guided EF-Tu library together with a Sec-specific selection system	Sec incorporation efficiency up to >90% and 2-fold increase in the yield of selenoprotein production [72]

Release factors	RF1	UAG	Bacteria	Use of antimicrobial peptides to temporarily inhibit the activity of RF1	>25-fold improvement in ncAA incorporation at multiple-sites [48]
	RF1	UAG	*E. coli*	Elimination of RF1 in *E. coli* strain with UAG-to-UAA synonymous changes in seven essential ORFs	48-fold improvement in protein yield with a single phosphoserine [81]
	RF1	UAG	*E. coli*	Removal of RF1 by fixing the RF2	Simultaneous incorporation of ncAAs at multiple sites [79]
	eRF1	UAG	*S. cerevisiae*	Transplanting TASNIKS and YCF motifs from *Tetrahymena* eRF1 into eRF1 of *S. cerevisiae*	~16-fold increase in readthrough of UAG [91]
	eRF1	UAG	Mammalian cells	Engineering of eRF1; best eRF1 variant (E55D)	5- to 7-fold improvement in ncAA incorporation [92]

Ribosome	16S rRNA	UAG	*E. coli*	Structure-guided A-site library with full random mutation for nucleotides (529–535) in the 530 loop of 16S rRNA	Improved efficiency of ncAA incorporation from ~20% to >60% and from <1% to >20% in response to one and two UAG codons, respectively [20]
	16S rRNA	UAG	*E. coli*	PACE-assisted directed evolution of 16S rRNA	~9-fold improvement in ncAA incorporation [103]
	23S rRNA	UUC	*E. coli*	Evolved ribosome mutant (P7A7) based on a previously discovered *β*-puromycin-sensitive ribosome mutant (040329)	3-fold improvement in *β*^3^-(p-Br)Phe incorporation in cells expressing P7A7 than 040329 [102]

### 3.1. Development and Optimization of Orthogonal aaRS/tRNA Pairs

Many orthogonal aaRS/tRNA pairs have been discovered and engineered for GCE. These pairs are often derived from phylogenetically distant organisms compared to the host of interest as the diverged identity elements of these orthogonal tRNAs prevent them from being recognized by endogenous aaRSs. For instance, *Methanocaldococcus jannaschii* tyrosyl-tRNA synthetase (*Mj*TyrRS)/*Mj*tRNA^Tyr^ pair is orthogonal in *Escherichia coli* (*E. coli*) [49]. The derivatives of the *E. coli Ec*TyrRS/*Ec*tRNA^Tyr^ pair and leucyl-tRNA synthetase (*Ec*LeuRS)/*Ec*tRNA^Leu^ pair could be utilized to incorporate various ncAAs in eukaryotic cells [50, 51]. The derivatives of pyrrolysyl-tRNA synthetase (PylRS)/tRNA^Pyl^ pairs are ideal for GCE. They have been extensively engineered as the PylRS/tRNA^Pyl^ pair is orthogonal in both bacteria and eukaryotic cells [2, 52]. Recent work created a series of chimeric aaRS/tRNA pairs that are orthogonal in both prokaryotic and eukaryotic cells by rationally transplanting the critical sequences from the PylRS/tRNA^Pyl^ pair into other canonical aaRS/tRNA pairs [53]. An integrated pipeline for discovering new orthogonal pairs was reported, which consists of both computational analysis and experimental validation [54].

Despite the successful development of many orthogonal aaRS/tRNA pairs, the mass production of ncAA-containing polypeptide remains a challenge. One major problem is the poor aminoacylation efficiency of modified orthogonal aaRSs. The structure-guided directed evolution strategy could create and improve the selectivity of orthogonal aaRS towards a designed ncAA, which relies on generating a library of mutants in the amino acid binding site of the orthogonal aaRS followed by successive rounds of positive selection (tolerance of antibiotics or fluorescence intensity) and negative selection (production of toxic proteins) [55]. Although the traditional strategy has been successfully applied at engineering aaRS for ncAA insertion, it is time-consuming and requires prior structural knowledge. To overcome these challenges, advanced methods have been developed to evolve aaRS/tRNA pairs. For instance, phage-assisted continuous evolution (PACE) and a simplified version termed phage-assisted noncontinuous evolution (PANCE) were used to generate highly active and selective aaRS variants by coupling random mutagenesis and delicate selection [56, 57]. Importantly, PACE and PANCE do not require the determined structure of aaRS to design the mutant library and thus have great potential for further evolving and improving orthogonal pairs. Multiplex automated genome engineering (MAGE) enables the generation of large libraries of chromosomal aaRSs by simultaneously mutagenizing different loci and producing polypeptide with 30 ncAA residues [58]. A recent study developed an integrated system called phage- and robotics-assisted near-continuous evolution (PRANCE) to optimize orthogonal aaRS/tRNA pairs, which offers several advantages, including scalability to high-throughput molecular experiments, a substantial reduction in reagents, and real-time feedback control [59].

tRNA is not a simple “generic adaptor.” Its optimization is often required for efficient and accurate biosynthesis of proteins containing ncAA. As many developed orthogonal tRNAs such as *Ec*tRNA^Tyr^ and tRNA^Pyl^ lack intrinsic A- and B-box elements that are important for RNA polymerase III-mediated transcription, heterologous expression of these tRNAs is problematic in eukaryotic cells. Improvement of cellular concentration of orthogonal tRNAs by promoter optimization [60, 61] and increase of tRNA copy number [62] are useful to enhance the production of ncAA-containing peptides and proteins. As tRNA has evolved to function with the ribosome and other translation factors in each organism [63], the introduced orthogonal tRNA might not function very well in heterologous hosts. Optimized tRNAs could be developed by enhancing their compatibility with translational apparatus via rational design and directed-evolution strategies [64–66]. In addition, tRNA modification is known to affect codon-anticodon pairing in the ribosome. However, the effect of tRNA modification on the performance of GCE remains poorly understood. Interestingly, the incorporation efficiency of *O*-phosphoserine (Sep) is affected by deletion and overexpression of some posttranscriptional modification enzymes in *E. coli* [67], highlighting the importance of tRNA modifications for genetically encoding ncAA in proteins.

### 3.2. Engineering of Elongation Factor

Although the formation efficiency of ncAA-tRNA could be significantly enhanced by optimization of orthogonal aaRS/tRNA pair, efficient delivery of charged tRNA by an elongation factor (*e.g.*, EF-Tu, the GTP-bound form of elongation factor in bacteria) to the ribosome remains as another challenge (Figure 1). EF-Tu recognizes both the acceptor helix of tRNA and amino acid moiety [68] and weakly binds negatively charged amino acids [1, 69]. Thus, EF-Tu often needs to be engineered to improve the incorporation efficiency of ncAAs with negative charges or bulky side chains. For instance, repurposing the substrate binding pocket of EF-Tu enables enhanced production of proteins containing ncAAs such as Sep, selenocysteine, and phenylalanine analogs, which are poorly incorporated by the wild-type EF-Tu [70–72]. Notably, the removal of EF-Sep21, a highly selective EF-Tu variant for Sep, abolishes the ability to genetically encode *O*-phosphoserine, highlighting the critical role of EF-Sep21 in incorporating negatively charged Sep [73].

### 3.3. Elimination and Engineering of Release Factors

Amber codon is commonly used for site-specific ncAA incorporation by GCE. Amber codon together with TAA (ochre stop) is recognized by release factor 1 (RF1), and RF2 recognizes TGA (opal stop) as well as ochre codon in *E. coli*. Thus, the amber codon-directed ncAA insertion by the orthogonal aaRS/tRNA pair competes with RF1-mediated translation termination, resulting in decreased production of ncAA-containing proteins (Figure 1). To eliminate the competition effect of RF1 and increase the efficiency of ncAA incorporation, many studies focused on the removal of RF1, a translation component that was thought to be essential [74, 75]. By expressing amber suppressor tRNA together with a few essential genes undergoing the stop codon swap (TAG>TAA), the RF1 could be deleted, and the amber codon was reassigned to encode ncAA [76]. Studies also found that RF1 is dispensable in various E. coli strains containing RF2 protein with higher activity than RF2(Thr246) in the K-12 strain [77–79]. Later construction of a GRO, in which the ochre codon replaces all instances of the amber codon, allows for the removal of RF1 and complete reassignment of TAG codon [5]. Alternatively, synonymous replacement of TAG in 95 genes of *E. coli* BL21(DE3) allows elimination of RF1, generating a robust growth strain [80]. Taking advantage of the RF1 knockout strain, efficient incorporation of ncAA at multiple UAG sites and improved phosphoserine and selenocysteine insertion were demonstrated [79, 81, 82]. The multisite incorporation of ncAA via cell-free translation system was also achieved by utilizing cell extracts of GRO lacking RF1 [83].

Unlike bacteria with separate RF1 and RF2, eukaryotic cells rely on a single omnipotent release factor (eRF1), an essential protein for cell viability, to recognize all stop codons [84]. Therefore, it is not feasible to improve the efficiency of ncAA in eukaryotic systems by simply eliminating eRF1. Inspired by findings that stop codons are reassigned invariant code organisms, tuning the specificity of eRF1 for stop codon recognition might be possible. For instance, TAG and TAA encode glutamine while translation termination relies on TGA in *Tetrahymena* and *Paramecium* [85, 86]. Biochemical and genetic studies identified several key residues in the N-terminal domain of eRF1 that are responsible for stop codon recognition [87–90]. Interestingly, the introduction of TASNIKS and YCF motifs from *Tetrahymena* eRF1 into eRF1 of *Saccharomyces cerevisiae* (*S. cerevisiae*) resulted in increased readthrough for amber codon [91]. Another study reported that ectopic overexpression of eRF1 mutant (E55D) enhances TAG-directed ncAA incorporation in mammalian cells [92]. Structural analyses also revealed the molecular basis of eRF1 recognition for stop codons [93, 94], which have implications for future structure-guided eRF1 engineering to enhance ncAA insertion by amber suppression.

### 3.4. Ribosome Engineering

A variety of *L-α*-amino acids with different noncanonical side chains has been genetically encoded in proteins [95], and certain types of *N*-methyl [96], D-*α*- [97], and *β*-amino acids [98] could be inserted into peptides by *in vitro* translation, indicating the substrate plasticity of native ribosomes. However, ribosome engineering is required to expand the substrate scope of translation, especially for those ncAAs with altered backbones. Utilization of ribosome mutants with modifications in the peptidyl transferase center (PTC), which catalyzes ribosome peptide bond formation and peptide release [99], could improve *in vitro* translation of dipeptides analogues and D- and *β*-amino acids [100, 101]. A recent study demonstrated cellular biosynthesis of a *β*-amino acid-containing DHFR by using *E. coli* cells expressing ribosome mutants together with wild-type EF-Tu and phenylalanyl-tRNA synthetase in the absence of phenylalanine [102]. Studies reported that ribosome mutants selected by randomization of 530 loop in 16S ribosomal RNAs (rRNA) and PACE-assisted directed evolution of 16S rRNA could enhance the efficiency of ncAA incorporation in living cells [20, 103].

## 4. Conclusion and Future Perspectives

The development of strategies to create orthogonal codons, optimize orthogonal translation components, and expand the substrate diversity of translation will enable and facilitate the cellular production and directed evolution of novel biopolymers. However, many challenges remain, including creating various functional chassis containing orthogonal codons and developing super active and orthogonal translation systems that can efficiently incorporate ncAAs into any POI regardless of context effects. In particular, efficient production of ncAA-containing proteins in eukaryotic cells is more challenging than that in the *E. coli* system since many approaches to creating blank codons are not easily transferred to eukaryotes and the development process of additional orthogonal aaRS/tRNA pairs is slow.

To promote future GCE application in eukaryotes, we propose yeasts, including *S. cerevisiae* and *Pichia pastoris*, could serve as powerful model systems since they are widely used in both high-through screening assays (*e.g*., yeast display) and production of recombinant POI for medical and industrial applications [104]. The ongoing synthetic yeast, Sc2.0, would open up new opportunities for GCE applications by creating an ideal eukaryotic chassis with the orthogonal codon [105]. Due to the TAG/TAA stop-codon swaps in the final Sc2.0 strain, it would be possible to safely engineer eRF1 for abolishing its recognition to the amber stop codon without causing unwanted global amber suppression. Thus, successful engineering of the eRF1 in Sc2.0 would ultimately generate a synthetic eukaryotic organism bearing an orthogonal codon for GCE. Furthermore, we think the cost reduction of genome writing and testing tools as well as the rapid development of technologies for yeast genome engineering would allow the reassignment of more blank codons, including stop and sense codons, to ncAAs by genome recoding of the synthetic yeast. Another key feature of Sc2.0 is the synthetic chromosome rearrangement and modification by loxP-mediated evolution (SCRaMbLE) system, which enables inducible whole-genome rearrangement by the Cre recombinase [106]. We think the utilization of the in-built SCRaMbLE system of Sc2.0 would be very powerful to generate cell populations with massive genomic diversity; the mutant strains showing improved GCE performance could be obtained by high-throughput selection method such as fluorescence-activated cell sorters (FACS). A previous work showed the disruption of the non-sense-mediated decay pathway by deleting the *UPF1* gene in *S. cerevisiae* could enhance the ncAA incorporation in response to the amber stop codon [107], highlighting the promise of genomic background modification to optimize GCE by SCRaMbLE.

To further create and optimize the orthogonal aaRS/tRNA pairs in various chassis in higher eukaryotes, continued efforts on the development of PylRS/tRNA^Pyl^ pair are worth adhering as this pair is orthogonal in both bacteria and eukaryotic cells. We recently disclosed all PylRS enzymes encoded in 524 archaeal genomes by mining the archaeal clusters of orthologous genes (arCOGs) [108]. We envision these PylRS/tRNA^Pyl^ pairs could be explored and engineered to enrich the toolbox for GCE. Taking the advantages of advanced methods for the directed evolution of biomacromolecules such as PACE, MAGE, and PRANCE, the efficiency and substrate specificity of PylRS/tRNA^Pyl^ pair candidates could be initially optimized in model organisms (*e.g*., *E. coli* and *S. cerevisiae*), followed by transplanting them into other eukaryotic cells. Scientists working with different eukaryotic chassis could then utilize their own expertise to further improve the expression level and intracellular compatibility of the introduced PylRS/tRNA^Pyl^ pairs. This concerted effort would provide guidance to optimize the process of developing super active and orthogonal aaRS/tRNA pairs for ncAA incorporation and lay a solid foundation for many exciting GCE applications in eukaryotes.
